# Supplementary material for: Planting date in South Kivu, eastern DR Congo: A real challenge for the sustainable management of Spodoptera frugiperda (Lepidoptera: Noctuidae) by smallholder farmers
Source: PLoS One. 2024 Dec 2;19(12):e0314615. doi: 10.1371/journal.pone.0314615 (PMC11611118; doi:10.1371/journal.pone.0314615)
Supplement: S1 Table — (DOCX) [file pone.0314615.s001.docx]

**S1 Table.** **Allocation of the number of fields being monitored according to planting dates, seasons and study locations**

| **Early season** | | | | | |
| --- | --- | --- | --- | --- | --- |
| **Planting date** | Timing | Miti-Murhesa | Katana | Mudaka | Total |
| 01 September 2020 | Early | 4 | 3 | 2 | 9 |
| 15 September 2020 | Early | 5 | 2 | 2 | 9 |
| 01 October 2020 | Early | 3 | 3 | 3 | 9 |
| 15 October 2020 | Late | 4 | 3 | 2 | 9 |
| 30 October 2020 | Late | 3 | 3 | 3 | 9 |
| **Total** | | **19** | **14** | **12** | **45** |
| **Late season** | | | | | |
| 01 February 2021 | Early | 3 | 3 | 3 | 9 |
| 15 February 2021 | Early | 4 | 2 | 3 | 9 |
| 01 March 2021 | Early | 4 | 3 | 2 | 9 |
| 15 March 2021 | Late | 3 | 4 | 2 | 9 |
| 30 March 2021 | Late | 3 | 3 | 3 | 9 |
| **Total** | | **17** | **15** | **13** | **45** |
