# Supplementary material for: Planting date in South Kivu, eastern DR Congo: A real challenge for the sustainable management of Spodoptera frugiperda (Lepidoptera: Noctuidae) by smallholder farmers
Source: PLoS One. 2024 Dec 2;19(12):e0314615. doi: 10.1371/journal.pone.0314615 (PMC11611118; doi:10.1371/journal.pone.0314615)
Supplement: S2 Table — (DOCX) [file pone.0314615.s002.docx]

**S2 Table.** **Summary of the results of the selection of Generalized linear mixed models (GLMMs) to explain the variability of larval density with other variables in early season**

| **Fixed effects** | **Model 1** | | | | | | | | | |
| --- | --- | --- | --- | --- | --- | --- | --- | --- | --- | --- |
|  | **Estimate** | **Std. Error** | **Z value** | **P value** | **AICc** | **AIC** | **BIC** | **logLik** | **Deviance** | **Df.resid** |
| **Intercept** | -1.414 | 1.04 | -1.34 | 0.177 | 302.32 | 285.8 | 312.9 | -127.9 | 255.8 | 30 |
| **Type of field (Exploitation)** | 0.547 | 0.34 | 1.58 | 0.112 |  |  |  |  |  |  |
| **Type of field (Farmer)** | 0.446 | 0.39 | 1.11 | 0.263 |  |  |  |  |  |  |
| **Surface (m^2^)** | -0.048 | 0.04 | -1.12 | 0.259 |  |  |  |  |  |  |
| **Planting time (Late)** | -0.045 | 0.16 | -0.28 | 0.779 |  |  |  |  |  |  |
| **Variety (M'Roma)** | 0.089 | 0.11 | 0.76 | 0.446 |  |  |  |  |  |  |
| **Variety (SAM4 Vita)** | 0.278 | 0.18 | 1.47 | 0.139 |  |  |  |  |  |  |
| **Variety (Z-M)** | 0.132 | 0.08 | 1.63 | 0.101 |  |  |  |  |  |  |
| **Fertilizers (Manure)** | -0.766 | 0.36 | -2.10 | **0.035** |  |  |  |  |  |  |
| **Fertilizers (None)** | -0.557 | 0.23 | -2.33 | **0.019** |  |  |  |  |  |  |
| **Fertilizers (NPK)** | -0.450 | 0.23 | -1.94 | 0.052 |  |  |  |  |  |  |
| **Fertilizers (Urea)** | -0.747 | 0.39 | -1.90 | 0.056 |  |  |  |  |  |  |
| **Fertilizers (Urea+Manure)** | -0.215 | 0.28 | -0.74 | 0.456 |  |  |  |  |  |  |
| **Julian calendar** | 0.016 | 0.00 | 4.14 | **< 0.001** |  |  |  |  |  |  |
| **Model 2** | | | | | | | | | | |
| **Intercept** | -1.17 | 0.52 | -2.21 | **0.026** | 297.86 | 283.9 | 309.2 | -127.9 | 255.9 | 31 |
| **Type of field (Exploitation)** | 0.56 | 0.33 | 1.7 | 0.089 |  |  |  |  |  |  |
| **Type of field (Farmer)** | 0.47 | 0.38 | 1.21 | 0.226 |  |  |  |  |  |  |
| **Surface (m^2^)** | -0.05 | 0.04 | -1.17 | 0.238 |  |  |  |  |  |  |
| **Variety (M'Roma)** | 0.09 | 0.11 | 0.77 | 0.439 |  |  |  |  |  |  |
| **Variety (SAM4 Vita)** | 0.28 | 0.18 | 1.5 | 0.133 |  |  |  |  |  |  |
| **Variety (Z-M)** | 0.13 | 0.07 | 1.79 | 0.073 |  |  |  |  |  |  |
| **Fertilizers (Manure)** | -0.78 | 0.35 | -2.19 | **0.027** |  |  |  |  |  |  |
| **Fertilizers (None)** | -0.58 | 0.22 | -2.64 | **< 0.01** |  |  |  |  |  |  |
| **Fertilizers (NPK)** | -0.47 | 0.21 | -2.26 | **0.023** |  |  |  |  |  |  |
| **Fertilizers (Urea)** | -0.78 | 0.37 | -2.10 | **0.035** |  |  |  |  |  |  |
| **Fertilizers (Urea+Manure)** | -0.23 | 0.28 | -0.82 | 0.411 |  |  |  |  |  |  |
| **Julian calendar** | 0.01 | 0.00 | 8.96 | **< 0.001** |  |  |  |  |  |  |
| **Model 3** | | | | | | | | | | |
| **Intercept** | -0.94 | 0.46 | -2.004 | **0.045** | 288.37 | 283.2 | 299.5 | -132.6 | 265.2 | 36 |
| **Surface (m^2^)** | -0.01 | 0.03 | -0.338 | 0.735 |  |  |  |  |  |  |
| **Fertilizers (Manure)** | -0.74 | 0.31 | -2.331 | **0.019** |  |  |  |  |  |  |
| **Fertilizers (None)** | -0.42 | 0.14 | -2.952 | **< 0.01** |  |  |  |  |  |  |
| **Fertilizers (NPK)** | -0.32 | 0.17 | -1.832 | 0.066 |  |  |  |  |  |  |
| **Fertilizers (Urea)** | -0.34 | 0.30 | -1.152 | 0.249 |  |  |  |  |  |  |
| **Fertilizers (Urea+Manure)** | -0.35 | 0.23 | -1.483 | 0.138 |  |  |  |  |  |  |
| **Julian calendar** | 0.01 | 0.00 | 9.432 | **< 0.001** |  |  |  |  |  |  |
| **Model 4** | | | | | | | | | | |
| **Intercept** | -0.91 | 0.46 | -1.97 | **0.047** | 285.34 | 281.3 | 295.8 | -132.7 | 265.3 | 37 |
| **Fertilizers (Manure)** | -0.73 | 0.31 | -2.32 | **0.020** |  |  |  |  |  |  |
| **Fertilizers (None)** | -0.41 | 0.13 | -2.97 | **< 0.01** |  |  |  |  |  |  |
| **Fertilizers (NPK)** | -0.33 | 0.17 | -1.88 | 0.059 |  |  |  |  |  |  |
| **Fertilizers (Urea)** | -0.34 | 0.30 | -1.15 | 0.250 |  |  |  |  |  |  |
| **Fertilizers (Urea+Manure)** | -0.33 | 0.23 | -1.44 | 0.148 |  |  |  |  |  |  |
| **Julian calendar** | 0.01 | 0.00 | 9.66 | **< 0.001** |  |  |  |  |  |  |
| **Model 5** | | | | | | | | | | |
| **Intercept** | -1.21 | 0.43 | -2.76 | **< 0.01** | 282.00 | 281.4 | 286.8 | -137.7 | 275.4 | 42 |
| **Julian calendar** | 0.01 | 0.00 | 10.04 | **< 0.001** |  |  |  |  |  |  |
