# Supplementary material for: Planting date in South Kivu, eastern DR Congo: A real challenge for the sustainable management of Spodoptera frugiperda (Lepidoptera: Noctuidae) by smallholder farmers
Source: PLoS One. 2024 Dec 2;19(12):e0314615. doi: 10.1371/journal.pone.0314615 (PMC11611118; doi:10.1371/journal.pone.0314615)
Supplement: S3 Table — (DOCX) [file pone.0314615.s003.docx]

**S3 Table. Summary of the results of the selection of Generalized linear mixed models (GLMMs) to explain the variability of larval density with other variables in late season**

| **Fixed effects** | **Model 1** | | | | | | | | | |
| --- | --- | --- | --- | --- | --- | --- | --- | --- | --- | --- |
|  | **Estimate** | **Std. Error** | **Z value** | **P value** | **AICc** | **AIC** | **BIC** | **logLik** | **Deviance** | **Df.resid** |
| **Intercept** | 3.38 | 0.46 | 7.22 | **< 0.001** | 335.50 | 321.5 | 346.8 | -146.7 | 293.5 | 31 |
| **Type of field (Exploitation)** | -0.27 | 0.33 | -0.81 | 0.416 |  |  |  |  |  |  |
| **Type of field (Farmer)** | -0.54 | 0.33 | -1.62 | 0.105 |  |  |  |  |  |  |
| **Surface (m^2^)** | -0.04 | 0.06 | -0.68 | 0.491 |  |  |  |  |  |  |
| **Planting time (Late)** | 0.56 | 0.14 | 3.76 | **< 0.001** |  |  |  |  |  |  |
| **Variety (M'Roma)** | 0.00 | 0.12 | 0.03 | 0.971 |  |  |  |  |  |  |
| **Variety (SAM4 Vita)** | 0.13 | 0.19 | 0.69 | 0.484 |  |  |  |  |  |  |
| **Variety (Z-M)** | -0.01 | 0.08 | -0.18 | 0.855 |  |  |  |  |  |  |
| **Fertilizers (None)** | -0.06 | 0.16 | -0.40 | 0.686 |  |  |  |  |  |  |
| **Fertilizers (NPK)** | -0.27 | 0.16 | -1.73 | 0.082 |  |  |  |  |  |  |
| **Fertilizers (NPK+Manure)** | -0.40 | 0.23 | -1.70 | 0.088 |  |  |  |  |  |  |
| **Fertilizers (Urea+Manure)** | -0.32 | 0.41 | -0.78 | 0.434 |  |  |  |  |  |  |
| **Julian calendar** | 0.00 | 0.00 | 0.18 | 0.855 |  |  |  |  |  |  |
| **Model 2** | | | | | | | | | | |
| **Intercept** | 2.72 | 0.43 | 6.33 | **< 0.001** | 345.71 | 334.0 | 357.5 | -154.0 | 308.0 | 32 |
| **Type of field (Exploitation)** | -0.10 | 0.33 | -0.31 | 0.750 |  |  |  |  |  |  |
| **Type of field (Farmer)** | -0.56 | 0.33 | -1.68 | 0.091 |  |  |  |  |  |  |
| **Surface (m^2^)** | -0.13 | 0.05 | -2.32 | **0.02** |  |  |  |  |  |  |
| **Variety (M'Roma)** | -0.14 | 0.12 | -1.21 | 0.222 |  |  |  |  |  |  |
| **Variety (SAM4 Vita)** | 0.12 | 0.19 | 0.65 | 0.510 |  |  |  |  |  |  |
| **Variety (Z-M)** | -0.10 | 0.08 | -1.25 | 0.208 |  |  |  |  |  |  |
| **Fertilizers (None)** | 0.02 | 0.15 | 0.14 | 0.887 |  |  |  |  |  |  |
| **Fertilizers (NPK)** | -0.32 | 0.15 | -2.00 | **0.044** |  |  |  |  |  |  |
| **Fertilizers (NPK+Manure )** | -0.42 | 0.23 | -1.78 | 0.074 |  |  |  |  |  |  |
| **Fertilizers (Urea+Manure)** | -0.32 | 0.41 | -0.77 | 0.440 |  |  |  |  |  |  |
| **Julian calendar** | 0.01 | 0.00 | 7.55 | **< 0.001** |  |  |  |  |  |  |
| **Model 3** | | | | | | | | | | |
| **Intercept** | 2.72 | 0.14 | 19.19 | **< 0.001** | 343.50 | 339.5 | 354.0 | -161.8 | 323.5 | 37 |
| **Surface (m^2^)** | 0.03 | 0.03 | 0.95 | 0.339 |  |  |  |  |  |  |
| **Fertilizers (None)** | -0.25 | 0.11 | -2.33 | **0.019** |  |  |  |  |  |  |
| **Fertilizers (NPK)** | -0.34 | 0.15 | -2.15 | **0.031** |  |  |  |  |  |  |
| **Fertilizers (NPK+Manure)** | -0.29 | 0.17 | -1.69 | 0.090 |  |  |  |  |  |  |
| **Fertilizers (Urea+Manure)** | -0.00 | 0.20 | -0.05 | 0.960 |  |  |  |  |  |  |
| **Julian calendar** | 0.01 | 0.00 | 7.46 | **< 0.001** |  |  |  |  |  |  |
| **Model 4** | | | | | | | | | | |
| **Intercept** | 2.72 | 0.14 | 19.15 | **< 0.001** | 341.43 | 338.4 | 351.1 | -162.2 | 324.4 | 38 |
| **Fertilizers (None)** | -0.27 | 0.10 | -2.52 | **0.011** |  |  |  |  |  |  |
| **Fertilizers (NPK)** | -0.33 | 0.15 | -2.11 | **0.034** |  |  |  |  |  |  |
| **Fertilizers (NPK+Manure)** | -0.31 | 0.17 | -1.79 | 0.073 |  |  |  |  |  |  |
| **Fertilizers (Urea+Manure)** | -0.06 | 0.19 | -0.33 | 0.739 |  |  |  |  |  |  |
| **Julian calendar** | 0.01 | 0.00 | 7.68 | **< 0.001** |  |  |  |  |  |  |
| **Model 5** | | | | | | | | | | |
| **Intercept** | 2.45 | 0.10 | 24.07 | **< 0.001** | 338.93 | 338.3 | 343.8 | -166.2 | 332.3 | 42 |
| **Julian calendar** | 0.01 | 0.00 | 8.07 | **< 0.001** |  |  |  |  |  |  |
